# The Home-Based Rehabilitation of Patients Through Physical Exercises in the Context of Indoor Air Quality

**DOI:** 10.3390/healthcare13131493

**Published:** 2025-06-23

**Authors:** Alexandru Bogdan Ilieș, Silviu Vlad, Tudor Caciora, Doriana Ciobanu, Dorina Ianc, Ana Cornelia Pereș, Thowayeb H. Hassan, Lazar Liviu

**Affiliations:** 1 Department of Surgical Specialties, Doctoral School of Biomedical Sciences, Faculty of Medicine and Pharmacy, University of Oradea, 410087 Oradea, Romania; ilies.alexandrubogdan@student.uoradea.ro; 2 Department of Surgical Specialties, Faculty of Medicine and Pharmacy, University of Oradea, 410087 Oradea, Romania; 3 Department of Geography, Tourism and Territorial Planning, Faculty of Geography, Tourism and Sport, University of Oradea, 1 Universitatii Street, 410087 Oradea, Romania; tudor.caciora@yahoo.com; 4 Department of Physical Education, Sport and Physiotherapy, Human Performance Research Center, University of Oradea, 410087 Oradea, Romania; dciobanu@uoradea.ro (D.C.); dianc@uoradea.ro (D.I.); 5 Department of Environmental Engineering, Faculty of Environmental Protection, University of Oradea, Magheru Street 26, 410087 Oradea, Romania; 6 Social Studies Department, College of Arts, King Faisal University, Al Hofuf 31982, Saudi Arabia; thassan@kfu.edu.sa; 7 Faculty of Medicine and Pharmacy, Doctoral School in Bio-Medical Science, University of Oradea, Str. Piața 1 Decembrie nr. 10, 410087 Oradea, Romania; lazarlv@yahoo.com

**Keywords:** indoor air quality, rehabilitation, physical exercises, spinal cord injury patients, indoor pollutants, air purification

## Abstract

**Background:** Patients with spinal cord injuries, in addition to rehabilitation in specialized facilities, often continue physical therapy at home. At that time, they become highly exposed to indoor pollutants, which can affect the effectiveness of the recovery program and human health. **Methods:** Thus, the present study presents the monitoring of indoor air quality in a residential facility where a patient with spinal cord injuries undergoes post-traumatic recuperative physical activity. Such a study is useful for ensuring good air quality for the optimal development of a rehabilitation program with the possibility of screening the indoor air quality of the home by the physiotherapist and even by the patient themselves, in the simplest way possible using low-cost equipment. Thus, 11 indoor air quality parameters were monitored for a period of 18 weeks, using low-cost equipment. An air purifier was put into operation for a period of one week to identify differences in the safety of the indoor environment for physical activities. **Results:** The results indicate an environment with frequent exceedances of the international standards in force for several indicators. After installing the purifier, the air quality stabilized and a much safer and more efficient environment for carrying out the recovery activities was established. **Conclusions:** Thus, the process of monitoring and optimizing indoor air quality stands as a fundamental requirement for home rehabilitation because it establishes a secure controlled environment that supports recovery in any residential setting.

## 1. Introduction

Patients with spinal cord injuries (SCIs) typically undergo complex physical rehabilitation programs in specialized medical facilities; however, to maintain therapeutic results, preserve muscle strength and flexibility, prevent muscle atrophy, and prevent secondary complications, it is essential to continue the recovery process through physical exercises carried out in a residential environment [1]. This component of rehabilitation is becoming increasingly important in the context of limited access to institutionalized therapies and the desire for the rapid reintegration of the patient into daily life [2,3,4]. At this stage, the patient spends long periods indoors, implicitly becoming more vulnerable to chronic exposure to indoor air pollutants. Thus, at this stage, the environment in which they carry out their recovery program must be as clean as possible [5]. During a physical exercise session, the respiratory system undergoes some physiological adaptations with the consequence of increasing the volume of inhaled air, and thus the quantity of pollutants inhaled in such an environment [6].

The respiratory function of patients with SCIs becomes impaired mainly when lesions occur at the upper thoracic or cervical levels because these areas have reduced lung capacity due to weakened respiratory muscles. This situation makes them more prone to hypoventilation, bronchial secretions accumulation, and recurrent respiratory infections. In this context, prolonged exposure to indoor air pollutants can worsen respiratory symptoms, decrease exercise tolerance, and delay recovery. Also, chronic inflammation induced by contaminants can interfere with the physiological adaptation processes to physical exercise, reducing the effectiveness of rehabilitation interventions. Thus, in the case of these patients, monitoring and improving the air quality of the indoor space where the physical exercise program is carried out becomes not only advisable but essential for the safety and efficiency of home recovery [7].

The main indoor air pollutants which people encounter most often include particulate matter (PM), carbon dioxide (CO_2_), total volatile organic compounds (TVOCs), formaldehyde (HCHO), and nitrogen dioxide (NO_2_), which negatively affect respiratory and cardiovascular health particularly when people perform intense physical activities [8,9,10]. Non-compliant values of temperature (T), relative humidity (RH), and inappropriate concentrations of positive (I^+^) and negative (I^−^) ions can have negative effects on well-being, cognitive capacity, and effort [11,12]. The main indoor air pollutants are generated directly by daily household activities, such as the use of cleaning products, personal care items, and room deodorizers, but also through emissions from construction materials, furniture, or vehicle infiltration, contributing significantly to TVOC and HCHO concentrations [13,14,15]. PM also comes largely from passive smoking, cooking processes, the use of heating systems, the burning of candles and insecticides, the presence of pets, and daily domestic activities [16]. In the absence of effective purification or filtration systems, the elimination of these pollutants depends mainly on natural or mechanical ventilation, which only partially contributes to reducing their concentrations in indoor spaces. In addition, the improper use of heating, ventilation, and air conditioning systems or such devices that are in improper working condition is also a contributing factor.

Studies in gyms have shown that elevated levels of PM and CO_2_ can exceed recommended values, especially under conditions of poor ventilation and high occupancy, negatively affecting both lung capacity and oxidative stress levels [17,18,19]. Consequently, an uncontrolled indoor environment can turn therapeutic exercise into an aggravating factor, undermining the benefits of rehabilitation and contributing to the onset of respiratory symptoms or general discomfort [20]. Other recent studies have highlighted that activities carried out in residential spaces can generate significant increases in indoor pollutant concentrations, especially PM, CO_2_, and TVOC, and especially in conditions of reduced ventilation or high furniture density [21,22,23]. Such conditions can amplify the exposure of individuals undergoing physical rehabilitation and undermine the benefits of therapy if not adequately controlled. The sympathetic nervous system decentralization in SCI patients severely damages vasomotor and sudomotor functions which makes thermal stress response inadequate [24]. Thermoregulatory capacity becomes severely impaired when patients perform physical exertion in hot or poorly ventilated environments, thus increasing their risk of developing hyperthermia, which demands immediate active cooling interventions and strict environmental management [25,26].

Although it is recognized that the environment in which rehabilitation takes place directly influences its effectiveness, indoor air quality (IAQ) is rarely systematically assessed in studies analyzing home rehabilitation [27,28]. Thus, the need for a multidisciplinary approach that includes, along with the physical component, also the optimization of IAQ conditions becomes evident, in order to support an efficient, safe, and sustainable recovery in a residential space. In a context where modern homes are increasingly airtight and daily activities contribute to the accumulation of indoor pollutants, the continuous monitoring of IAQ parameters becomes a necessity to protect the health of occupants, especially in the case of vulnerable people or those undergoing medical recovery. Multiple studies have highlighted that the use of real-time monitoring systems, based on multiparametric sensors and advanced technologies, allows for not only the prompt detection of harmful values, but also effective intervention through automatic purification systems or appropriate corrective measures [7,18,29,30,31,32]. Various studies have considered IAQ monitoring and indoor–outdoor environment correlations, as well as IAQ during physical exercise (especially concentrations of CO_2_, CO, PM, NO_2_, etc.) which have an impact on health and require interventions to improve IAQ [8]. Therefore, the implementation of intelligent air detection and filtration solutions becomes a fundamental condition for maintaining a safe, healthy indoor environment compatible with physical activity or therapeutic recovery.

Given the growing body of evidence linking IAQ to health and rehabilitation outcomes, a systematic investigation is needed to assess the real-world conditions encountered by patients during home-based recovery. Addressing this knowledge gap can help establish evidence-based recommendations and practical interventions that enhance both safety and therapeutic efficacy in domestic rehabilitation environments.

However, the current literature remains relatively limited in terms of applied and integrated approaches investigating IAQ in the real homes of patients undergoing physical rehabilitation, especially in the context of long-term, multifactorial monitoring directly correlated with concrete technological interventions such as air purification. Thus, given the increased vulnerability of SCI patients to IAQ variations, as well as the documented influence of IAQ parameters on respiratory function, thermoregulation, and exercise tolerance, the working hypothesis of this study was that monitoring and optimizing IAQ through the use of accessible technologies and targeted interventions can significantly contribute to creating a favorable environment for the safe and effective implementation of physical rehabilitation exercises at home.

In this context, the study aimed to determine the real values of the IAQ parameters in the home of a patient in a home recovery program; the extent to which these values fall within the limits recommended by international standards for a healthy indoor environment; the impact the use of a high-performance air purifier has on the levels of the monitored pollutants; and whether this intervention can be considered an effective, replicable, and easy-to-implement solution in other similar contexts of residential medical rehabilitation. The novelty of this study lies in the integrated approach to IAQ in the context of physical rehabilitation carried out at home through the extensive monitoring of a wide range of physical and chemical pollutants, corroborated via the assessment of the impact of a concrete technological intervention. In the absence of specific standards for residential recovery spaces, the study proposes a feasible and reproducible model for evaluating and optimizing IAQ for vulnerable patients.

## 2. Materials and Methods

### 2.1. Case Study

The apartment in which the study was conducted consists of two rooms, with an area of 44 m^2^, and is located on the 1st floor of a cultural heritage building (Ullmann Palace), a representative building of the Viennese Secession style, built in 1913, in the Municipality of Oradea, Romania. This apartment is inhabited by a patient with SCI who must carry out a rehabilitation program at home consisting of physical activities in order to recover from post-traumatic injuries. At the time of the research, the building was in an advanced state of structural degradation, with rehabilitation works planned as part of conservation and restoration projects for local architectural heritage. The location of the apartment in a historic building with thermal insulation deficiencies, limited mechanical ventilation, and potential passive sources of pollutants specific to old materials provided a real context for IAQ assessment under the conditions of a home medical rehabilitation program.

### 2.2. Indoor Air Quality Monitoring

The first stage of the study was the determination of IAQ using manual and datalogger sensors. Thus, the determination of T, RH, CO_2_, TVOC, HCHO, I^+^, and I^−^ concentrations and PM of different sizes (PM_1_, PM_2.5_, PM_4_, and PM_10_) was considered. In the context of physical rehabilitation at home, these IAQ indicators were selected because they directly influence thermal comfort, respiratory function, and exposure to pollutants [33]. Thus, they were monitored to capture the combined impact on patient health and the efficiency of the recuperative therapy carried out in the indoor environment. The monitoring took place over a period of 18 weeks, between 6 December 2024 and 11 April 2025.

**Figure 1:**
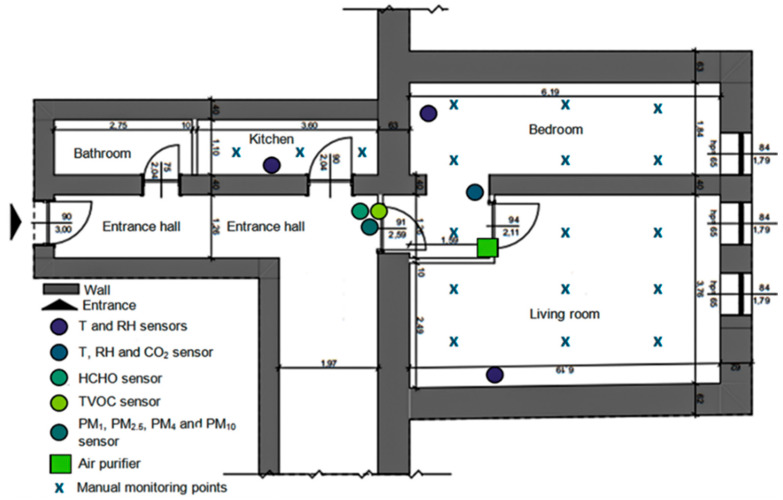
Spatial distribution of IAQ sensors and data collection points.

The above-mentioned indicators were determined in the most important rooms of the apartment (the living room, bedroom, and kitchen), where the patient spends most of the day and where (especially the living room) he carries out his recovery program as indicated by the doctor. The datalogger systems, for determining T, RH, CO_2_, TVOC, HCHO, PM_1_, PM_2.5_, PM_4_, and PM_10_, were placed in such a way as to capture the distribution of these IAQ indicators throughout the apartment, being mounted in its center, between the three rooms considered. The exception were the sensors for determining T and RH, which were mounted in each of the three rooms (Figure 1).

Three HOBO U23 Pro v2 devices (Onset Computer Corporation, Bourne, MA, USA) were dispersed across the room’s surface to accurately determine T and RH values. The U23 is a datalogger with a T measurement resolution of 0.02 °C at 25 °C and an accuracy of ±0.21 °C from 0 °C to 50 °C; RH is determined with a resolution of 0.05% and an accuracy of ±2.5% from 10% to 90%, up to a maximum of ±3.5%, as well as 5% above 90% and below 10%. The device operates as an offline datalogger which prevents real-time cloud data transmission and lacks display functionality, requiring specialized software to view recorded data.

An Extech SD800 datalogger (Extech Instruments, Nashua, NH, USA) operated in the analyzed room to monitor T and RH values and CO_2_ concentration simultaneously. The technical specifications of this device show a ±0.8 °C accuracy and a 0.1 °C resolution for T measurements, a ±4% accuracy and a 0.1% resolution for RH measurements, a 1 ppm resolution with a ±40 ppm accuracy for CO_2_ measurements below 1000 ppm, and a ±5% accuracy for CO_2_ measurements above 1000 ppm. A single device operated in the room to monitor CO_2_ concentrations because this gas poses health risks to humans when found in poorly ventilated closed spaces.

The determination of the amount of suspended particles was performed using a Delta OHM HD50PM device (Delta OHM Srl, Padova, Italy). This is a professional web datalogger device designed for monitoring PM of different sizes in real time. The device uses an optical laser sensor to measure PM_1_, PM_2.5_, PM_4_, and PM_10_ concentration levels. The device provides a 0.1 μg/m^3^ resolution and shows a ±10 μg/m^3^ accuracy for 0…100 μg/m^3^ measurements and a ±10% accuracy for 100…1000 μg/m^3^ measurements.

The concentration of TVOC and HCHO was determined using a fixed gas detector E2632 (Evikon MCI, Tartu, Estonia) designed for the simultaneous monitoring of two parameters of the chemical contamination of indoor air by integrating two independent detection channels. The technical specifications show HCHO detection between 0 and 5 ppm with a response time of less than 120 s and a ±5% accuracy for the measured values. The TVOC sensor measures between 0 and 500 ppm with a response time of less than 120 s and a ±15% accuracy for the measured values.

For PM_1_, PM_2.5_, PM_4_, PM_10_, CO_2_, TVOC, and HCHO concentrations, as well as for determining T and RH values, the devices used were set to retrieve and store values every hour. This approach aimed to achieve an optimal balance between the temporal granularity of the data and the operational feasibility of long-term monitoring. This approach was essential for the precise identification of the moments and circumstances of the occurrence of anomalies, providing a relevant framework for correlating environmental factors with the patient’s daily activities and living conditions. In addition, the hourly interval facilitates comparability between parameters and allows for the detection of chronological trends without statistical distortions.

In addition to the automated datalogger systems, manual sensors were also used in the study to cover both existing technological limitations and to increase the accuracy of the data obtained. Thus, parameters such as I^+^ and I^−^ were monitored exclusively by manual sensors, in the absence of datalogger equipment available for continuous measurements, and for PM_2.5_ and PM_10_ particulate matter, the manual sensors were used complementarily to capture point variations and validate automatic recordings, thus contributing to a more accurate characterization of IAQ in the context of home rehabilitation.

Regarding the determination of I^+^ and I^−^, this was performed with a handheld device NKMH-103 (Nippon Keiryoki Manufacturing Co., Ltd., Tokyo, Japan). It displays in real time both the I^+^ and I^−^ concentrations, with a sensitivity of up to 10 ions/cm^3^, a resolution of 1 ion/cm^3^, and an accuracy of approximately ±10% for the measured value. For the monitoring of PM_2.5_ and PM_10_ concentrations, a PCE-PCO 2 device (PCE Instruments, Southampton, UK) was used. It has a resolution of 1 µg/m^3^ and a measuring range between 0 and 1000 µg/m^3^, with a typical accuracy of ±10%, making it suitable for the rapid and efficient assessment of particulate pollution in indoor spaces. Using these devices, between one and three daily measurements were performed, within 18 collection points distributed in the three analyzed rooms (Figure 1). The ultimate goal was to generate a sufficiently consistent and representative database, capable of accurately reflecting the real variability of IAQ parameters in daily living and activity conditions.

**Figure 2:**
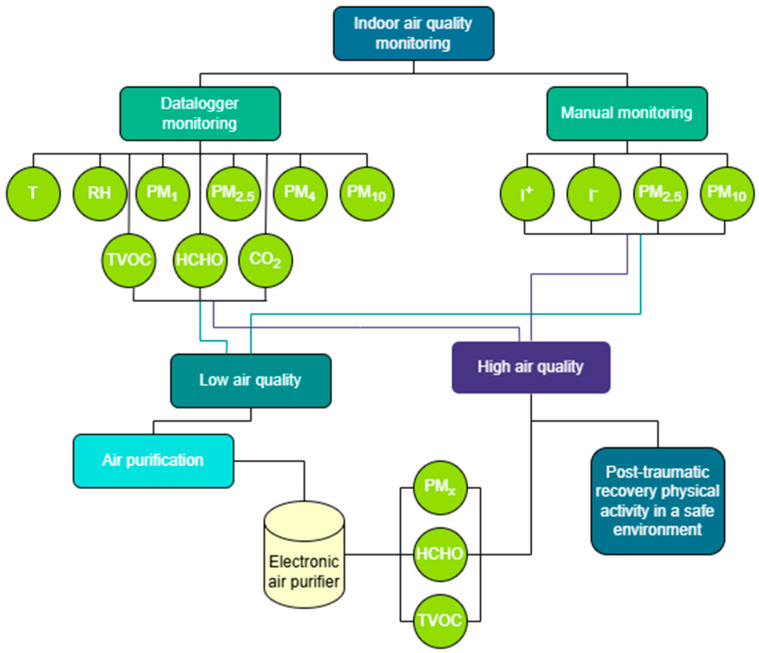
The methodological stages of conducting the study.

The second stage of the study involved installing a Dyson Purifier Cool For-maldehyde TP09 (Dyson Limited, Malmesbury, Wiltshire, UK) air purification device to evaluate its effectiveness in maintaining a clean and stable indoor environment suitable for home-based physical rehabilitation activities. The Dyson TP09 features a three-stage filtration system which includes a catalytic filter, a HEPA H13 filter, and activated carbon to capture 99.97% of particles ≥0.3 µm and convert formaldehyde into water and carbon dioxide. The device features a high-precision solid-state sensor which detects formaldehyde molecules down to 500 times smaller than 0.1 µm while maintaining detection accuracy throughout its operational life (Figure 2).

### 2.3. Interventions

The intervention aimed to quantify the impact of the device on the concentrations of chemical pollutants and suspended particles, in order to optimize IAQ in residential spaces used for therapeutic purposes. It is designed to eliminate PM (PM_1_, PM_2.5_, PM_4_, and PM_10_), TVOC, HCHO, NO_2_, allergens, dust, bacteria, and odors. The device combines a HEPA H13 filter with an activated carbon filter and a special catalyst for the continuous decomposition of HCHO. The device was centrally located, in the common distribution area of the apartment, and operated continuously during week 17 of the monitoring period. Subsequently, a comparative analysis of the concentrations for the monitored pollutants (PM_1_, PM_2.5_, PM_4_, PM_10_, TVOC, and HCHO) was carried out in order to evaluate the direct impact of using the purifier on IAQ by comparing it to the values recorded in its absence (Figure 2).

The researchers deliberately chose to implement the air purification intervention during week 17. The researchers needed 16 weeks of pre-intervention time to create a complete baseline record of IAQ variability under normal, unaltered environmental conditions. The extended monitoring period allowed for the documentation of pollutant concentrations, indoor activity patterns, and seasonal environmental influences before the intervention began. The extended temporal framework strengthens both the reliability and statistical power of subsequent comparisons, improving the internal validity of the effects caused by the purification system.

### 2.4. Statistical Analysis

The values obtained for pollutants were reported to the international standards in force regarding IAQ and exposure to various pollutants in the context of human health, set by organizations such as the World Health Organization (WHO), the US Environmental Protection Agency (EPA), and the European Union. The collected data were subsequently processed and interpreted using specialized statistical analysis software applications in order to rigorously evaluate variations in the monitored parameters and identify any significant deviations from the reference standards.

The evaluation of indoor air quality changes after the air purification intervention used paired two-tailed Student’s *t*-tests for each monitored parameter. The statistical analysis used dependent (paired) samples because measurements were taken before and after the intervention in the same location. The test determines if the pre- and post-intervention mean differences differ significantly from zero. The two-tailed statistical test was used to evaluate the possibility of both parameter value increases and decreases. Each air quality indicator received time-synchronized measurements under controlled conditions using identical instruments at the same sampling points to ensure data consistency and reduce external variability. The test produced t-statistics along with degrees of freedom and *p*-values for each measured parameter. The calculation of Cohen’s d provided effect size estimates to evaluate the practical significance of observed changes in addition to statistical significance.

## 3. Results

According to the most recognized international guidelines on comfort and health conditions in residential spaces, the optimal indoor T should be between 20 and 24 °C in the cold season and 23–26 °C in the hot season, and the RH should be maintained in the range of 30–60%, to ensure both thermal comfort and the prevention of the development of microorganisms or the occurrence of respiratory irritations, as recommended by ASHRAE [34] and the World Health Organization [35]. Also, the minimum T in homes should not fall below 18 °C, especially in the case of vulnerable people or people with chronic conditions, a category that also includes patients in physical rehabilitation.

**Figure 3:**
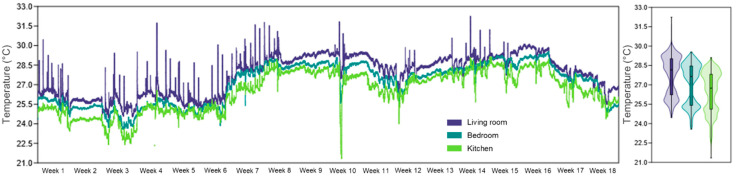
Evolution of T during the 18 weeks of monitoring, in the three analyzed rooms of the apartment, highlighting internal thermal variations in the context of IAQ assessment associated with physical rehabilitation at home.

The thermal comfort measurements showed high values across all examined areas, which surpassed the recommended upper limit for moderate activity levels. The living room displayed the highest temperature readings with 27.7 °C, followed by the bedroom at 27.1 °C and the kitchen at 26.4 °C (Figure 3). The living room showed 100% of measured values above the threshold while the bedroom data exceeded the threshold in 98.3% of cases and the kitchen data showed the lowest values with 94.7% above the threshold. The elevated T values create substantial negative effects on patients with neurological conditions including SCI because these patients commonly experience impaired thermoregulation abilities.

**Figure 4:**
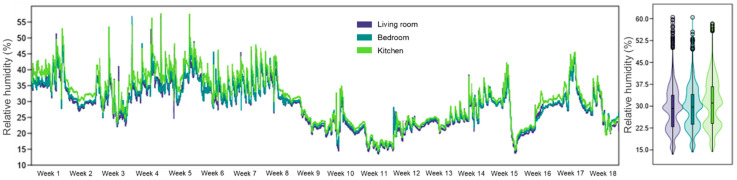
RH evolution during the 18 weeks of monitoring, in the three analyzed rooms of the apartment, highlighting the internal RH variations in the context of IAQ assessment associated with physical rehabilitation at home.

The RH measurements stayed lower than the 40–60% range which defines healthy indoor air quality throughout all three rooms. The living room maintained an average relative humidity of 28.7%, the bedroom had an average relative humidity of 29.3%, and the kitchen 30.8% (Figure 4). The recorded values exceeded international standard ranges by approximately 51.1%, with the living room showing the highest percentage of 58.4% of data points.

The high T values and low RH levels observed in all of the analyzed rooms can significantly reduce the efficiency of home physical rehabilitation and negatively impact the overall health of patients with SCI.

**Figure 5:**
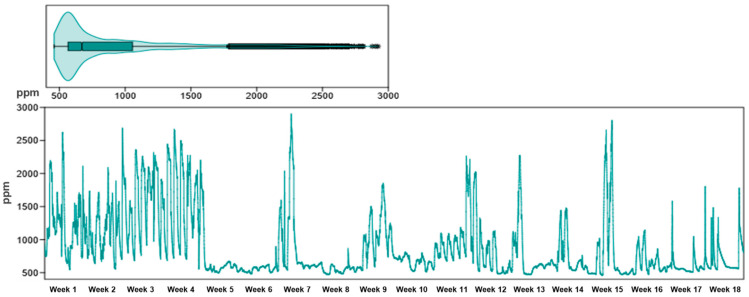
Evolution of CO_2_ during the 18 weeks of monitoring, in the three analyzed rooms of the apartment, highlighting the variations in this indicator in the context of the IAQ assessment associated with physical rehabilitation at home.

According to the ASHRAE standards [36], widely used for IAQ regulation, a CO_2_ concentration of less than 1000 ppm is considered an indicator of adequate ventilation and an acceptable level of indoor air comfort and hygiene. The CDC and NIOSH [37] also specify that the level of 1000 ppm is comfortable for workplaces and the upper limit for occupational exposure during a working day (8 h) is 5000 ppm. The CO_2_ concentration in the monitored apartment was 893 ppm on average (Figure 5; Table 1). Average levels around the 900 ppm threshold indicate insufficient ventilation in certain intervals, and peaks above 2000 ppm suggest excessive CO_2_ accumulation during periods of intense activity or a lack of ventilation. At the same time, 27.5% of the recorded values exceeded international standards, which can induce feelings of fatigue, headache, difficulty concentrating, and decreased cognitive performance; aspects particularly relevant in the case of patients undergoing rehabilitation, where mental efficiency and exercise tolerance need to be optimized (Norbäck et al., 2013) [38].

Air ionization is an essential indicator of IAQ, with a direct impact on respiratory health, general well-being, and performance during physical activities. In the absence of rigorous international standards, the literature recommends that the number of I^+^ in indoor air should not exceed 1000 ions/cm^3^, as high concentrations are frequently associated with adverse health effects, such as irritability, fatigue, headache, or respiratory discomfort; on the other hand, I^−^ should be above the threshold of 1000 ions/cm^3^, being correlated with beneficial effects on the psychophysiological state, including improved breathing, mood, and reduced oxidative stress [39,40,41]. Thus, maintaining a favorable ratio between I^+^ and I^−^ is an important criterion in the assessment of IAQ, especially in the case of spaces intended for rehabilitation activities.

**Figure 6:**
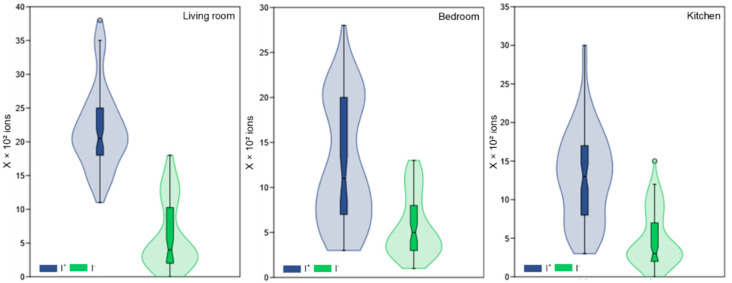
The concentration of I^+^ and I^−^ inside the three rooms monitored over a period of 18 weeks.

The living room recorded an average of 2180 ions/cm^3^ for I^+^ and 601 ions/cm^3^ for I^−^. On the other hand, in the bedroom, 1253 ions/cm^3^ were recorded on average for I^+^ and 556 ions/cm^3^ for I^−^, while in the kitchen, the average values were 1300 ions/cm^3^ for I^+^ and 484 ions/cm^3^ for I^−^ (Figure 6; Table 1).

The recorded values are below the reference thresholds as per the literature. About 63.7% of the values measured for I^+^ exceeded the permissible limit of 1000 ions/cm^3^ and 85.7% of the values for I^−^ did not reach the threshold of 1000 ions/cm^3^ set by the international standards. This indicates that the air in the room is weakly negatively ionized which can be detrimental to the performance of physical exercises and the well-being of the patient.

On the IAQ in living spaces, international standards set very specific limits for TVOC that may affect human health. For TVOC, the recommended values state that the annual average should be less than 1 mg/m^3^ according to the WHO and European Union guidelines. and levels above 3 mg/m^3^ are considered alert levels for short-term exposure. For the extreme toxic thresholds, they are set at approximately 590 mg/m^3^, values that are not commonly encountered but present severe acute risk [42,43]. The World Health Organization (WHO) sets the maximum recommended limit for HCHO at 0.1 mg/m^3^ (~0.08 ppm) for short-term exposure because this concentration protects against respiratory irritation and systemic effects [42].

**Figure 7:**
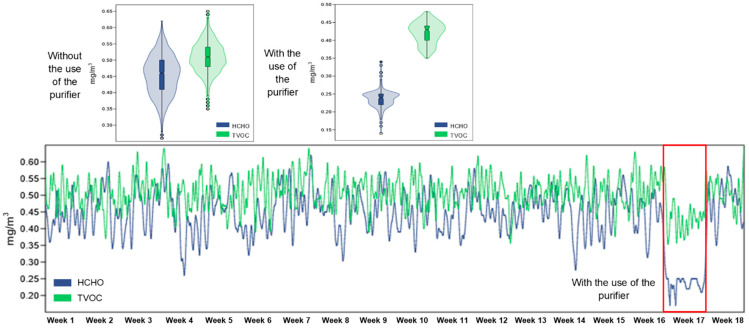
The evolution of the concentration of HCHO and TVOC inside the three rooms monitored over a period of 18 weeks, as well as the difference between the period in which the air purifier was used and the periods in which it was not used.

The HCHO concentration values during the monitoring period were found to be significantly different based on the presence or absence of the air purifier. Without the device, the mean HCHO concentration was 0.453 mg/m^3^, the highest was 0.622 mg/m^3^, the lowest was 0.261 mg/m^3^. When the purifier was put into operation, the values significantly decreased, the average being 0.236 mg/m^3^, the maximum was 0.335 mg/m^3^, and the minimum was 0.140 mg/m^3^ (Figure 7; Table 1). This difference shows that the average concentration of HCHO was reduced by approximately 47.8%, and this is because all the recorded values are above the permissible values. Such a decrease in HCHO concentration can contribute to the development of a safer and healthier environment for physical activity.

During the test period, in the absence of the purifier, the average TVOC concentration was 0.509 mg/m^3^. After activating the purifier, the values decreased, with the average concentration reaching 0.424 mg/m^3^, with a maximum of 0.502 mg/m^3^ and a minimum of 0.352 mg/m^3^ (Figure 7; Table 1). All these values are within the permissible limits, and this reduction corresponds to an average decrease of approximately 16.7%, indicating the moderate but clear effectiveness of the intervention in limiting exposure to volatile chemical pollutants.

The WHO Global IAQ Guidelines [44] identifies PM as a dangerous air pollutant which causes respiratory and cardiovascular diseases and premature death. The updated standards set a maximum annual average of five µg/m^3^ for PM_2.5_ and 15 µg/m^3^ for PM_10_. The WHO sets 15 µg/m^3^ as the 24 h threshold for PM_2.5_ and 45 µg/m^3^ for PM_10_ to protect vulnerable populations from acute risks. The guideline does not establish direct standards for PM_1_ and PM_4_ fractions but experts consider them more dangerous because of their small size and deep respiratory tract penetration and the literature suggests maintaining them below 10–15 µg/m^3^ in indoor spaces [45,46].

**Table 1:** Descriptive statistics of indoor air quality parameters measured in relation to applicable international standards.

Indicator	Sample Size	MU	Min	Avg	Max	std. dev.	AIS	Ref-AIS
T	18,288	°C	21.3	27.1	32.2	1.6	20–24 °C cold season, 23–26 °C hot season	[34,35]
RH	18,288	%	13.6	29.5	60.5	7.2	30–60%	[34,35]
CO_2_	18,288	ppm	460	893	2920	485	1000 ppm	[36,37]
HCHO	18,288	mg/m^3^	0.14	0.44	0.62	0.078	<0.1 mg/m^3^	[42]
TVOC	18,288	mg/m^3^	0.35	0.5	0.65	0.05	<1 mg/m^3^	[42,43]
PM_1_	18,288	µg/m^3^	6.9	14.9	20.1	2.25	NSI	
PM_2.5_	18,288	µg/m^3^	6.1	15.7	21.4	2.43	15 µg/m^3^	[45,46]
PM_4_	18,288	µg/m^3^	6.4	15.6	20.7	2.58	NSI	
PM_10_	18,288	µg/m^3^	6.7	15.7	20.7	2.42	45 µg/m^3^	[45,46]
I^−^	311	no. × 10^3^	0	5.5	18	4	>10 × 10^3^ ions	[39,40,41]
I^+^	311	no. × 10^3^	3	15.7	38	7.5	<10 × 10^3^ ions	[39,40,41]

MU—measure unit, Min—minimum absolute value, Max—maximum absolute value, Avg—average value for entire apartment, std. dev.—standard deviation, AIS—applicable international standards; NSI—not standardized internationally; Ref-AIS—references for AIS.

As part of the comparative IAQ assessment, PM levels of various sizes (PM_1_, PM_2.5_, PM_4_, and PM_10_) were analyzed in the absence and presence of the dedicated air purifier. The results obtained highlight significant reductions in average concentrations for all monitored PM categories, confirming the effectiveness of the device in limiting indoor environmental pollution.

**Figure 8:**
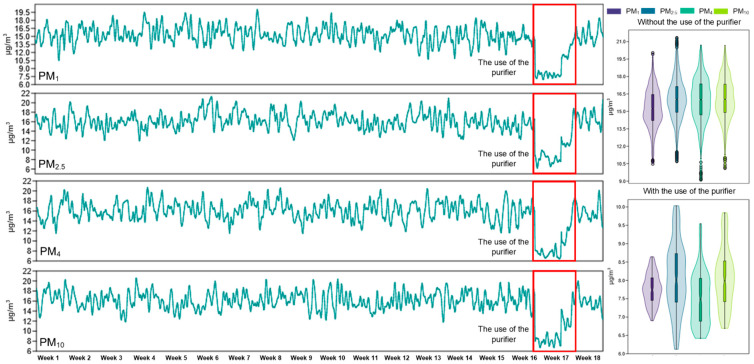
The evolution of the concentration of PM_1_, PM_2.5_, PM_4_, and PM_10_ inside the apartment was monitored over a period of 18 weeks, as well as the difference between the period in which the air purifier was used and the periods in which it was not used.

The ultrafine particle (PM_1_) concentration decreased to 7.8 µg/m^3^ after purifier installation from its initial value of 15.3 µg/m^3^, which resulted in a 49.2% reduction and the standard deviation decreased from ±1.6 µg/m^3^ to ±0.4 µg/m^3^, indicating better indoor air quality stability. The PM_2.5_ values decreased from 16.1 µg/m^3^ to 8.1 µg/m^3^ which resulted in a 49.7% reduction and the variability decreased from ±1.8 µg/m^3^ to ±1 µg/m^3^. The PM_4_ concentration showed the biggest reduction at 53% as the mean value dropped from 15.9 µg/m^3^ to 7.5 µg/m^3^ with standard deviations of ±1.9 µg/m^3^ and ±0.7 µg/m^3^, respectively. The PM_10_ mean concentration decreased to 8.1 µg/m^3^ from 16.1 µg/m^3^, which resulted in a 49.8% reduction, while the standard deviation decreased from ±1.8 µg/m^3^ to ±0.8 µg/m^3^ (Figure 8; Table 1).

All measured values dropped below the 10 µg/m^3^ limit established by international standards after purifier installation, which demonstrates the effective filtration capability of the purifier and its positive impact on IAQ. The improvement in air quality is vital for human health because PM exposure leads to respiratory inflammation and worsens chronic lung diseases and reduces exercise tolerance according to Zhang et al. [47].

Although the majority of the evaluated parameters showed statistically significant differences, the changes observed in some of them were not abrupt or impactful. This may indicate that their values are not strongly dependent on the purification intervention.

The most relevant air quality indicators, HCHO and TVOC, recorded the most notable decreases. In the case of HCHO, the difference between the means was ΔM = 0.191, with a 95% confidence interval (0.184, 0.198), t value = 53.49, and *p* < 0.0001. The effect size calculated by Cohen’s d = 5.697 indicates the very large impact of the intervention. Similarly, for TVOC, ΔM = 0.079, t = 18.95, and *p* < 0.0001, with a Cohen’s d = 2.074, also in the area of very strong effects. The values recorded for suspended particles PM_1.0_, PM_2.5_, and PM_4.0_ also showed highly significant decreases. For PM_1.0_, the *t*-test indicated t = 50.81, *p* < 0.0001, and Cohen’s d = 6.073, reflecting a massive reduction in particulate matter. PM_2.5_, a clinically relevant parameter for respiratory health, recorded t = 55.34, *p* < 0.0001, and Cohen’s d = 6.615, while PM_4.0_ had t = 45.09, *p* < 0.0001, also with a very large effect (d = 5.390) (Table 2). These results demonstrate that the air purification intervention was highly effective in significantly reducing concentrations of HCHO, TVOC, and PM. The observed reductions are particularly relevant from a health perspective, as these pollutants, especially PM, are known to penetrate deep into the respiratory tract and contribute to various adverse health outcomes, including respiratory and cardiovascular diseases.

For other parameters, such as T, RH, CO_2_, I^+^, and I^−^, no statistically significant differences were observed, the effect size being small, suggesting a weaker influence or natural variations within the room.

## 4. Discussion

The evaluation of the IAQ indicators revealed that multiple parameters surpassed international standards for human activity spaces including T, RH, CO_2_, TVOC, HCHO, PM_1_, PM_2.5_, and PM_10_. The exceedances become crucial for home physical rehabilitation because physical exercise enhances pulmonary ventilation, leading to higher pollutant inhalation doses that negatively affect respiratory health and cardiovascular and cognitive functions [48]. Recent studies show that exercise in polluted air conditions worsens airway inflammation, reduces lung capacity, and promotes oxidative stress, especially in vulnerable individuals [49,50]. Also, elevated PM concentrations can trigger bronchospasm and alterations in blood pressure, especially among people with respiratory allergies [51]. It has been shown that even in apparently protected enclosed spaces, such as homes, TVOC, CO_2_, and PM concentrations can exceed safe values, with direct effects on cognitive function, mood, and the level of oxygen available to tissues during exercise [52]. In addition, increased CO_2_ concentrations in poorly ventilated spaces have been correlated with decreased cognitive performance and the occurrence of respiratory discomfort during exercise [53]. All these studies support the assertion that monitoring and optimizing IAQ is an essential condition for the safety and efficiency of physical recovery at home.

The air purifier installation in the monitored apartment resulted in major reductions in PM concentrations together with HCHO and TVOC levels, which are harmful to respiratory and general health. The patient’s home-based physical rehabilitation activities increase exposure to contaminants because these pollutants need to be reduced in this specific environment. Multiple recent studies have demonstrated that air purifiers successfully decrease PM and TVOC levels, which leads to beneficial outcomes for airway inflammation, lung function, and oxidative stress. Research has demonstrated that HEPA purifiers enhance cardio-respiratory biomarkers in healthy individuals [54] and these devices lead to major PM_2.5_ reductions and better respiratory health in homes where children have allergies [55]. Research data demonstrates that PM and TVOC concentrations decrease by more than 90% in both laboratory and field environments [56,57]. The use of purifiers has been linked to decreased blood pressure and inflammatory markers in people who experience severe urban pollution [58].

To further reduce the health risk to the patient performing physical activities indoors, careful regulation of T and RH within recommended comfort ranges (22–24 °C and 40–60%) is essential, as extreme values can compromise physiological performance and increase susceptibility to infections and mucosal irritation [59,60]. Frequent ventilation by opening windows or applying mechanical solutions is recommended to eliminate excess accumulated CO_2_, a factor associated with decreased cognitive performance and respiratory discomfort [61]. In addition, the use of I-generating devices can further contribute to improving IAQ, neutralizing volatile pollutants, and supporting general well-being [62,63]. Integrating these strategies into an IAQ control protocol is especially recommended in the homes of patients with sustained physical activity, to reduce health risks and support the efficiency of rehabilitation.

The results obtained in this study are consistent with those reported in the literature, which highlight the effectiveness of air purifiers equipped with HEPA filters in reducing PM and VOC in indoor spaces inhabited by people with special needs or vulnerable people [64,65]. In addition, our methodological approach, based on continuous multiparametric monitoring in a real residential setting, complements other studies that have focused on controlled laboratory environments or short-term interventions, adding applicability to home rehabilitation practice [66]. These findings support the usefulness of integrating such accessible and effective solutions into home rehabilitation protocols, contributing to creating an environment conducive to long-term functional recovery.

Although the results confirm known conclusions regarding the effectiveness of air purifiers, the study adds value by applying it to a less standardized but clinically relevant home-based recovery context. The selection of a patient with SCI, a vulnerable and rarely addressed category in this type of research, emphasizes the importance of personalized environmental monitoring. The system used is simple, affordable, and replicable, providing a concrete basis for future studies investigating the direct impact of air quality on functional recovery.

## 5. Conclusions

The present study demonstrates the importance of monitoring and optimizing IAQ in the context of home-based physical rehabilitation, especially for patients with SCI, who are highly vulnerable to environmental factors. The results obtained from the extensive monitoring over a period of 18 weeks revealed frequent exceedances of the optimal values for T, CO_2_, TVOC, HCHO, and PM, as well as a significant deficit of RH and I^−^, all of which can negatively affect the efficiency and safety of physical exercises. The use of an air purifier led to a significant reduction in the concentrations of HCHO, TVOC, and PM, highlighting the positive impact of the technology on IAQ. In addition, the reduced variability of the values after the installation of the device suggests an effective stabilization of the environment. By integrating accessible equipment and a feasible monitoring protocol, the study proposes an applicable and replicable solution for improving home rehabilitation conditions. It is therefore recommended to develop good practice guidelines that include IAQ assessment and control as an integral part of therapeutic programs for chronic patients treated in residential settings. At the same time, the results of this study highlight the fact that, by using low-cost monitoring equipment, the patient or physiotherapist can actively participate in IAQ management, quickly intervening on environmental parameters through ventilation, humidity adjustment, or the activation of purification systems, which significantly contributes to maintaining an optimal framework for carrying out physical rehabilitation outside specialized centers.

Beyond the obvious advantage of low costs, the solution proposed in this study offers multiple practical benefits—the monitoring systems used are easy to install and operate even by the patient or therapist, without requiring advanced technical training; can be adapted to various home configurations; and allow for rapid intervention depending on variations in environmental parameters. These features transform the proposed model into a scalable solution that is easy to replicate in other home rehabilitation contexts, thus contributing to increasing the accessibility and efficiency of the therapeutic act.

## 6. Limitations and Future Perspectives

A limitation of the study is that the analysis was conducted in a single apartment and for a single patient, which limits the generalizability of the results. Also, the particular architectural conditions of the monitored building (a historical building in a state of degradation) may influence pollutant concentrations and ventilation levels differently than modern homes, which requires caution in extrapolating the conclusions.

Future monitoring will also target some comfort and thermoregulation parameters in patients with SCI who carry out physical rehabilitation programs in an environment with a high degree of pollution, also monitoring the influence that the environment has on the efficiency of recuperative physical exercises. At the same time, the air humidifier used in the study does not include T and RH regulation functions, nor the capacity for negative ionization of indoor air, and in the absence of a functional HVAC system in the analyzed apartment, these technical limitations affect the complete control of IAQ; consequently, future studies will aim at integrating complex equipment, capable of simultaneously regulating these essential parameters for an optimal rehabilitation environment.

## Figures and Tables

**Table 2:** The results of the independent samples *t*-test on the differences between air quality parameters before and after the purifier intervention.

Indicator	Mean-BF	Mean-AF	ΔM	Variance-BF	Variance-AF	t	*p*-Value	Cohen’s D
T	26.8	28.4	1.63	19.95	22.81	7.63	3.6958 × 10^−14^	0.353
RH	21.7	27.3	5.6	2.21	16.06	18.3	8.4651 × 10^−55^	1.83
CO_2_	624	589	35	28,906	15,447	5.11	3.4051 × 10^−07^	0.237
HCHO	0.428	0.237	0.191	0.0016	0.0007	53.49	5.957 × 10^−174^	5.697
TVOC	0.503	0.423	0.079	0.0016	0.0012	18.95	8.576 × 10^−55^	2.074
PM_1_	14.88	7.74	7.13	2.58	0.17	50.81	9.7341 × 10^−143^	6.073
PM_2.5_	15.83	8.09	7.75	1.83	0.9	55.34	4.0209 × 10^−152^	6.615
PM_4_	15.21	7.5	7.7	3.58	0.5	45.09	7.1052 × 10^−130^	5.39
PM_10_	16.74	8.07	8.66	3.04	0.66	53.35	4.4247 × 10^−148^	6.377
I^−^	5.45	5.48	0.027	15.35	16.98	0.05	0.959	0.006
I^+^	15.17	16.31	1.15	64.94	45.62	1.13	0.257	0.154

BF—before air purification; AF—after air purification.

## Data Availability

The raw data supporting the conclusions of this article will be made available by the authors without undue reservation.

## Abbreviations

The following abbreviations are used in this manuscript:

SCI	spinal cord injury
IAQ	indoor air quality
PM	particulate matter
TVOC	total volatile organic compounds
HCHO	formaldehyde
T	temperature
I^+^	positive ion concentration
I^−^	negative ion concentration
EPA	US Environmental Protection Agency
HVAC system	heating, ventilation, and air conditioning system
